# Perceived stigma and associated factors among family caregivers of patients with severe mental disorders

**DOI:** 10.1192/j.eurpsy.2024.1259

**Published:** 2024-08-27

**Authors:** R. Masmoudi, W. Abid, E. Ben salem, R. Ouali, I. Feki, I. Baati, J. Masmoudi

**Affiliations:** psychiatry A department, Hedi chaker university hospital, Sfax, Tunisia

## Abstract

**Introduction:**

Giving care to someone who is experiencing mental distress is a difficult and challenging task that could be detrimental to the caregiver’s overall quality of life. Stigma associated with mental illness is one of the most important mental health issues faced by these caregivers.

**Objectives:**

Our aims were to assess perceived stigma among family caregivers of patients with severe mental disorders and to identify its associated factors.

**Methods:**

We conducted a descriptive and analytical cross-sectional study among family caregivers of patients followed at the psychiatry outpatient clinic of the Hedi Chaker University Hospital in Sfax, during the period from February 2022 to July 2022.

A structured interview questionnaire was designed to collect socio-demographic data of both patients and their caregivers. We used the Stigma Devaluation Scale (SDS) to assess stigma.

**Results:**

A total of 90 family caregivers of severely mentally ill patients were included: 26 men and 64 women, with an average age of 50.68 ±11.67 years.

Patients’ parents accounted for 40% of family caregivers. The majority of family caregivers (83.3%) had no more than secondary education. Married people represented 70% of cases.

The median age of patients was 42 years. Schizophrenia was the diagnosis in 68.9% of cases. The mean duration of illness was 16.23 years.

Daily assistance lasted from 4 to 8 hours in 30% of cases and more than 8 hours in 66.7% of cases.

The mean score (SDS12) for family-focused stigma was 13.12 ± 2.34 with ranges from 8 to 18.

Perceived stigma scores were significantly higher among caregivers caring for non-married patients (p=0.04), with an age <50 years (p=0.04), and with a higher level of education (p=0.02).

Long duration of providing care (> 8 hours per day) (p=0.05) and insufficient information about the illness (p=0.02) were significantly associated with perceived stigma.

**Conclusions:**

The clinicians managing patients with severe mental disorders must focus on stigma and psychological distress among the caregivers and plan intervention strategies to reduce stigma.

**Disclosure of Interest:**

None Declared

